# Lipopolysaccharide-anchored macrophages hijack tumor microtube networks for selective drug transport and augmentation of antitumor effects in orthotopic lung cancer

**DOI:** 10.7150/thno.37380

**Published:** 2019-09-21

**Authors:** Ling Guo, Ye Zhang, Runxiu Wei, Cuifeng Wang, Min Feng

**Affiliations:** School of Pharmaceutical Sciences, Sun Yat-sen University, University Town, Guangzhou, 510006, P.R. China

**Keywords:** engineered macrophage, tumor microtube network, tumor tropism, TNF-α release, orthotopic lung cancer

## Abstract

**Objective:** Engineered immune cells (e.g., therapeutic T cells) provide a revolutionary approach to combat cancer. Certain activated immune cells can exquisitely sense and respond to the tumor microenvironment. Here, we propose a paradigm based on engineering macrophages to allow selective intercellular drug delivery and augmentation of antitumor activities by hijacking tumor microtube networks.

**Methods:** Macrophages were engineered *via* anchoring lipopolysaccharides on the plasma membrane (LM). The tumor tropism of LM encapsulating doxorubicin (LM-Dox) was monitored by a real-time cell migration assay and small animal *in vivo* imaging. Monocyte chemoattractant protein-1 (CCL2) was measured by quantitative PCR and ELISA. Intercellular conduit formation was characterized by confocal laser scanning microscopy and scanning electron microscopy. LM-Dox activation of tumor-associated macrophages to release TNF-α was evaluated by western blot and immunofluorescence assays. The potential therapeutic effects of LM-Dox in a 3D tumor-immune model and a murine orthotopic lung cancer model were tested.

**Results:** LM-Dox exhibited tumor tropism in response to CCL2 produced by A549 lung tumor cells and lung tumor tissues resulting in a remarkably higher amount of tumor accumulation than the case of Lipo-Dox (~ 4-fold). Intriguingly, LM-Dox accumulated at tumor sites hijacked the established tumor microtube networks and even stimulated microtube formation with tumor cells but not with normal cells to enable selective and rapid transport of the drug to tumor cells. Simultaneously, LM-Dox induced secretion of TNF-α in tumor-associated macrophages, which increased the antitumor activity of Dox. Thus, LM-Dox increased the inhibitory effects on tumor growth and metastasis in a mouse orthotopic lung cancer model and minimized the side effects of Dox-induced tumor invasion.

**Conclusion:** Lipopolysaccharide-anchored macrophages that can hijack tumor microtube networks for selective drug transport may serve as versatile bioactive carriers of anticancer drugs. In the clinical context, these engineered microphages represent a personalized medicine approach that can be translated into potential use of patient-derived monocytes/macrophages for drug delivery by means of cell-to-cell communication.

## Introduction

The emergence of engineered living cells (e.g., therapeutic T cells) as a form of cancer treatment provides a revolutionary approach to combat cancer 1. Furthermore, live cells surmounting physiological barriers to drug transport mark the beginning of a new era in drug delivery systems. Cells substantially differ from the inanimate drug delivery platforms, such as polymeric nanoparticles and liposomes, because live cells can exquisitely sense and respond to tumor microenvironment. For example, tumor cells and stroma produce monocyte chemoattractant protein-1 (CCL2), which is a major chemoattractant for monocytes/macrophages 2. Macrophages are the effector cells of the innate immune system and express C-C chemokine receptor type 2 (CCR2) in response to CCL2 resulting in continuous recruitment of macrophages to the tumor sites. 3, 4. Moreover, it is well known that administration of nanoparticles into biological fluids results in the formation of a protein corona *via* the adsorption of proteins on nanoparticles; this process subsequently influences blood circulation and tumor-targeting capacity 5-7. However, macrophages are phagocytic cells that are able to engulf and digest anything that does not have certain specific types of proteins characteristic for healthy cell surface 8. Thus, macrophages, acting as drug delivery carriers, may be able to remove adsorbed proteins *via* phagocytosis to overcome the impact of the protein corona on targeting capacity of drug carriers. These characteristics of macrophages make them attractive carriers for anticancer drug delivery.

Lipopolysaccharide (LPS) is a macromolecule composed of lipids and polysaccharides and is present in the outer membrane of gram-negative bacteria 9. LPS can interact with CD14 and Toll-like receptor 4 (TLR4), which are expressed on macrophages and are the high-affinity receptors for LPS 10. LPS has been shown to reprogram tumor-associated macrophages (TAMs) to the M1-like phenotype in a TLR4-dependent manner 11. TAMs usually comprise M2-polarized macrophages and are associated with protumor features, whereas M1-like macrophages have antitumor functions 12. TAM shift toward M1-like macrophages is considered an efficient means to promote tumor regression by secretion of inflammatory cytokines with potent antitumor activity, including tumor necrosis factor α (TNF-α) 13. It has been shown that the effects of TNF-α are synergistic with various anticancer drugs, such as doxorubicin (Dox), etoposide, and actinomycin D 14, 15. Thus, we rationalized engineering of macrophages by anchoring LPS on the plasma membrane to enhance the anticancer activity of encapsulated drugs.

Numerous malignant tumors have a higher proportion of stromatous cells than that of tumor cells. Thus, tumor cells are not in a constant immediate physical contact and are separated by the extracellular matrix components 16. The spatially separated tumor cells form the tumor microtube networks to maintain their long-range contact detected in lung cancer, ovarian cancers, osteosarcomas, breast cancers, neuroendocrine tumors, and colon cancers 17. Furthermore, relatively recent results indicate that cellular tumor microtube networks are significantly upregulated in tumor cells compared to that in stromal or non-malignant cells 18 because cancer cells divide quickly and survive under the conditions of physiologic and metabolic stress, including nutrient stress, oxidative stress, hypoxia, etc. 19. Thus, rapid proliferation of the cancer cells requires continuous exchange of information and biosynthetic materials between them *via* tumor microtubes 20. Macrophages can form intercellular conduits 21 and tumor microtubes connect tumor cells to each other in cancer patients with various cancer types; hence, we hypothesized that LPS-anchored macrophages may hijack the established intercellular tumor microtube networks in the tumor tissues to specifically deliver therapeutic agents to tumor cells for the antitumor effects. Here, we show that systemic administration of LPS membrane-anchored macrophages encapsulating Dox (LM-Dox) to mice with orthotopic lung cancer inhibits the growth of primary and metastatic cancers. We provide evidence that this effect is due to tumor tropism *via* hijacking the tumor microtube network for selective drug transfer, induction of TNF-α release, and suppression of Dox-induced tumor invasion resulting in massive tumor cell apoptosis and superior survival benefits.

## Materials and methods

### Materials

Doxorubicin hydrochloride (DoxHCl) was purchased from Meilun Biology Technology Co., Ltd (Dalian, China). Lipophilic doxorubicin (Dox) was prepared by desalting DoxHCl into the nonprotonated form. The commercial product of Lipo-Dox is a liposomal formulation resembling DOXIL^®^ (Doxorubicin Hydrochloride Liposome Injection, manufactured by China Shijiazhuang Pharmaceutical Group Co., Ltd., Batch No: H20113320). RPMI-1640 medium, Dulbecco's Modified Eagle Media (DMEM) medium, Opti-MEM^®^ I Reduced Serum medium, fetal bovine serum (FBS), trypsin and penicillin/streptomycin were obtained from Gibco (Canada). Annexin V-FITC apoptosis detection kit was purchased from Bestbio Biology (Shanghai, China). TRIzol reagent, Lipofectamine 3000 transfection reagent and the far-red fluorescent, lipophilic carbocyanine DiD were the products of Invitrogen (USA). Plasmid mEGFP-Lifeact-7 was ordered from Addgene (USA). 4′, 6-Diamidino-2-phenylindole dihydrochloride (DAPI), dimethyl sulfoxide (DMSO), 3-(4,5-dimethylthiazol-2-yl)-2,5-diphenyl tetrazolium bromide (MTT), LPS-FITC and BCA Protein Quantitation Assay Kit were obtained from Sigma-Aldrich (USA). 2.5% glutaraldehyde for SEM and 4% paraformaldehyde solution were supplied by Leagene Biotechnology (Beijing, China). RNAiso Plus (Total RNA extraction reagent), PrimeScript™ RT reagent Kit with gDNA Eraser (Perfect Real Time) and TB Green™ Premix Ex Taq™ II (TliRNaseH Plus) were purchased from Takara Bio (Shiga, Japan). The high-affinity F-actin probe Alexa Fluor^®^ 647 phalloidin and Anti-rabbit IgG, HRP-linked antibody were the products of Cell Signaling Technology (USA). RIPA lysis buffer, Phenylmethanesulfonyl fluoride (PMSF), primary/secondary antibody dilution buffer and loading buffer were purchased from Beyotime Biotechnology (Shanghai, China). Immobilon Western chemiluminescent HRP substrate and Polyvinylidene fluoride (PVDF) membranes were purchased from Millipore (USA). Anti-mannose receptor antibody (rabbit, ab64693), anti-liver arginase antibody (rabbit, ab91279) and anti-β tubulin antibody (rabbit, ab6046) were supplied by Abcam (Britain). TNF-α polyclonal antibody was supplied by Bioworld Technology (USA).

### Cell lines and animals

A murine macrophage cell line RAW264.7, a human non-small cell lung cancer cell line A549, a A549/GFP (GFP, green fluorescent protein) human non-small cell lung cancer cell line stably expressing GFP, a human lung fibroblast cell line HLF were purchased from the American Tissue Culture Collection. All cells were kept in a 37 °C humidified incubator (Thermo, U.S.A) with 5% CO_2_. Female BALB/c nude mice (four weeks old) were purchased from the Laboratory Animal Center, Sun Yat-sen University (Guangzhou, China) and kept under SPF conditions, with ready access to standardized food and water. All the animal experiments conducted were strictly observed the Guiding Principles for the Use of Laboratory Animals and were approved by the Institutional Animal Care and Use Committee of the Sun Yat-sen University.

### Preparation of LPS-anchored macrophages (LM) and Dox-loaded LM (LM-Dox)

LPS-anchored macrophages of the subtype M1 were prepared by the coincubation method. RAW264.7 cells were cultured in DMEM containing 10% fetal bovine serum with 1.0 μg/ml of LPS (L3024, Sigma-Aldrich, USA) and 20 ng/ml of IFN-γ (315-05, PeproTech, USA) for 48 h. The preparation procedure was performed in a 37 °C humidified incubator under the atmosphere of 5% carbon dioxide. LPS is able to interact with CD14 and Toll-like receptor 4, which are expressed on macrophages and are the high-affinity receptors for LPS.

The nonprotonated Dox was prepared by desalination of DoxHCl according to a modified method. Briefly, DoxHCl (20 mg, 0.0345 mmol) was dissolved in dichloromethane containing triethylamine (14.3 µl, 0.1032 mmol). The mixture was continuously stirred for 24 h at room temperature and then dialyzed to remove unreacted DoxHCl, triethylamine and triethylamine hydrochloride. Lipophilic Dox was obtained as a dark red powder after lyophilization.

Dox-loaded macrophages were obtained by incubating LM or inactive macrophages with Dox. Briefly, LM or inactive macrophages (1 × 10^5^ cells/mL) were seeded in a sterile tube. After culture in the FBS-free medium for 1 h, macrophages were incubated with lipophilic Dox at a concentration of 300 nmol/mL at 37 °C for 2 h. After washing with 5% glucose solution thrice to remove free Dox, the LM-Dox or M-Dox suspension was obtained. Dox loading profiles were analyzed by flow cytometry (EPICS XL, Beckman Coulter, USA). LM+Dox and M+Dox were prepared by physically mixing Dox and macrophages immediately prior to use.

### Confocal imaging

RAW264.7 cells were grown to confluence on coverslips and were incubated with LPS-FITC (1 μg/mL) for 48 h at 37 °C. Then, plasma membrane was visualized by lipophilic carbocyanine dye DiD and nuclei were stained with DAPI. Images of the samples were recorded under a laser scanning microscope (FV3000, Olympus, Japan).

For intercellular conduit observation, 1 ×10^5^ A549 cells were seeded on a glass-bottomed Petri dish and 1 ×10^4^ LM-Dox were added to coculture for 2 h. Then, the cells were washed three times with HBSS, stained with high-affinity F-actin probe Alexa Fluor® 647 phalloidin for 15 min, and mounted on glass slides using Prolong Gold antifade reagent (Molecular Probes). Cells were visualized by a laser scanning microscope.

### Live-cell fluorescence imaging

A549 cells (1×10^5^) were seeded on a glass-bottomed Petri dish and subjected to lipoplex-mediated transfection for 24 h;, the transfection solution was prepared by gently mixing 2.5 μg of pDNA (mEGFP-Lifeact-7) and 3.75 μL Lipofectamine 3000 solution in Opti-MEM^®^ I reduced serum medium and incubation at room temperature for 30 min. Then, 1×10^4^ LM-DiD were added. Images of the samples were recorded under a laser scanning microscope.

### *In vitro* tumor tropism

Tropism assays were carried out in the CIM-16-well plates using an xCELLigence RTCA-DP instrument (Roche Diagnostics, UK). Supernatant of A549 cell culture or normal cell HLF culture was collected after 48 h and diluted with RPMI 1640 medium to desired concentrations. Then, the supernatants were loaded into the lower wells of the CIM-16 plate. Upper chamber was attached and upper well was filled with 30 μL prewarmed medium; the plate was pre-equilibrated for 30 min. LM-Dox and M-Dox were resuspended to 2×10^5^ cells/mL and 100 μL cell suspension (2×10^4^ cells) was placed into each of the top wells. The assembled plate was transferred to the RTCA-DP machine and the data were collected every 5 min over the course of 240 sweeps (20 h in total). Electrical impedance signal corresponds to the cell index as shown in a representative trace. Increase in cell impedance correlates to an increase in the number of migrated macrophages adhering to the bottom of the lower chamber.

### *In vitro* anti-invasion activity

Cell invasion tests were carried out in the CIM-16-well plates; each well consists of an upper chamber and a lower chamber separated by a microporous membrane containing randomly distributed 8-μm pores. xCELLigence RTCA-DP instrument was used to record the data (Roche Diagnostics). The system measures a dimensionless parameter called cell index, which evaluates the ionic environment at an electrode/solution interface and integrates the information on cell numbers. The complete cell culture medium was loaded into the lower wells of the CIM-16 plate. Upper chamber was attached and coated with Matrigel to equilibrate at 37 °C for 4 h. To initiate an experiment, 100 μL of cell suspension was seeded into the wells (10,000 cells per well). After cell addition, CIM-16 plates were incubated for 30 min at room temperature in a laminar flow hood to allow the cells to settle onto the membrane according to the manufacturer's guidelines. Then, the drugs were added. Each condition was tested in duplicate with a programmed signal detection every 5 min during 7 h of incubation. The data were recorded by the supplied RTCA software (vs. 1.2.1). To analyze the results, the cell index values of the selected wells at 1 h were set to a constant (Delta Constant) with a default value of one.

After the cell invasion tests, the CIM-16-well plates were removed from the instrument and the membranes were cut, fixed with 2.5% glutaraldehyde and processed for scanning electron microscopy.

### Western blot analysis

Mannose receptor (CD206), arginase-1 and TNFα expression levels in tumor-associated macrophages (TAMs) were detected by denaturing nonreducing SDS-PAGE. Cells were lysed with RIPA-buffer (20 mM Tris, pH 7.5, 150 mM NaCl, 0.5 mM EDTA-2Na, 1% sodium deoxycholate, 0.1% sodium dodecyl sulfate, 1% IGEPAL supplemented with Complete Mini EDTA-free protease inhibitors (Roche Applied Science) and 1% phosphatase inhibitor cocktails 2 and 3 (P5726 and P0044, Sigma Aldrich)), scraped off after 5 min on ice and centrifuged at 14,000 g for 20 min at 4 °C. SDS-polyacrylamide gel electrophoresis was carried out with 20-35 μg of whole cell lysate from each sample. The gels were blotted onto a polyvinylidene difluoride membrane (Millipore) and blocked for 1 h at room temperature. Primary antibodies were added and membranes were incubated overnight at 4 °C. After washing three times with TBST buffer, HRP-conjugated secondary antibodies diluted 1:3,000 in blocking buffer were added and the blots were incubated for 1 h at room temperature. Blots were scanned using a gel imaging system (4600, Tanon, China). Western blots were quantified using ImageJ (NIH, USA). The background of the blots was subtracted by the rolling ball subtraction method with a radius of 50 pixels. Then, the integrated intensity of the bands was measured.

### Immunofluorescence assay

TAMs were seeded on glass coverslips in 24-well plates for 24 h prior to the experiments. After treatment with LM-Dox, M-Dox or Lipo-Dox (equivalent to 8 µM Dox) for another 24 h, cells were fixed in 4% paraformaldehyde for 15 min at room temperature, permeabilized with 0.1% Triton X-100 for 15 min and blocked with 3% BSA for 30 min. Then, cells were incubated overnight at 4 °C with an appropriate primary antibody of TNF-α. After washing three times with PBST, cells were incubated with goat anti-mouse Alexa Fluor 546 for 1 h in the dark at room temperature. Cell nuclei were stained by Hoechst (Sigma-Aldrich Corporation). Samples were observed under a laser scanning microscope.

### RNA preparation and real-time quantitative PCR

TAMs were incubated with TAK242 (1 μM, TLR4 inhibitor) for 1 hour. LM-Dox were treated with PMB (10 μg/mL, LPS inhibitor) for 30 min. Then, Dox, M-Dox and LM-Dox (equivalent to 8 µM of Dox) were added. Total RNA in TAMs was extracted from 5 × 10^6^ cells using RNAiso Plus (TaKaRa) and was reverse-transcribed into cDNA using the PrimeScript RT Reagent Kit (TaKaRa) according to the manufacturer's instructions. Prepared cDNA was then subjected to quantitative PCR analysis. A Bio-Rad MyiQ Real-Time PCR Detection System (Bio-Rad Laboratories) was used to measure SYBR Green (IQ SYBR Green Supermix, Bio-Rad Laboratories) incorporation with the following protocol: 95 °C for 15 s, 40 cycles of 94 °C for 10 s, 60 °C for 30 s, 72 °C for 30 s. Data acquisition was performed during this 72 °C extension step. Melting curve analysis was performed from 72 to 95 °C. The change in cycling threshold (ΔΔCt) method was used to analyze levels of transcripts and data were normalized to the level of GAPDH. The primers were as follows: TNF-α: GCGACGTGGAACTGGCAGAAG (for), GAATGAGAAGAGGCTGAGACATAGGC (rev); CCL2: AGAATCACCAGCAGCAAGTGTCC (for), TTGCTTGTCCAGGTGGTCCATG (rev); CCL3: CCGGTGTCATCTTCCTAACCAAGC (for), TCAGGCACTCAGCTCCAGGTC (rev); M-Sec: CTGGAGGTGGTGGTGGAGAGG (for), CAGAGCAGCAGCAAGTAGGTATCC (rev).

### Enzyme-linked immunosorbent assay (ELISA)

CCL2, TNF-α and IL10 were measured by ELISA kits as described by the manufacturer. Supernatants were diluted 2- to 10-fold for ELISA analysis. Serum samples were diluted 1:5 for the TNF-α assay. All samples were analyzed in duplicate.

### 3D multicellular tumor-immune spheroid model

3D tumor-immune spheroid model consisting of macrophages and tumor cells was prepared in 96-well low-attachment culture plates following the manufacturer's instruction. When the cells reached approximately 80% confluence, they were harvested by trypsinization and resuspended to get single-cell suspension. To generate multicellular spheroids, the mixture of 100 RAW264.7 cells and 500 tumor cells in 100 μL of culture media was seeded to 96-well low-attachment culture plates and the plates were incubated for 72 h in a 37°C humidified incubator with 5% CO_2_ until spheroids formed. The successful construction of 3D tumor-immune spheroid model was determined by light microscopy of hematoxylin and eosin (H&E)-stained sections. After subsequent drug treatments, a digital inverted microscope (EVOS, Fisher Scientific, U.S.A) was applied to monitor the spheroid formation and growth. Cell viability was measured using a Calcein-AM/Propidium iodide double staining kit (YEASEN Biotechnology, China) based on manufacturer's protocol. Live cells were identified by the conversion of the cell permeant non-fluorescent Calcein-AM to the fluorescent calcein dye by intracellular esterase activity, while dead cells with the permeabilized membrane were stained with membrane-impermeable fluorescent dye propidium iodide (PI) at 96 h after exposure to drugs. The relative fluorescence intensities of the red-fluorescent (PI, λ_ex_ 535 nm, λ_em_ 617 nm) dead cells versus the green-fluorescent (Calcein-AM, λ_ex_ 490 nm, λ_em_ 515 nm) live cells in spheroids were used to evaluate anti-tumor activities. Quantification analysis of signal intensity was determined with ImageJ software (NIH). The spheroids volumes were estimated from the major (a) and minor (b) axes using the following formula: a × b^2^ × 0.5.

### Dox transfer to tumor cells

1 × 10^5^ cells/well was seeded in a 12-well dish for 24 h prior to each experiment. LM-Dox (0.250 pmol/macrophage; 1.6×10^4^ macrophages/mL equivalent to 4 µM of Dox), M-Dox (0.159 pmol/macrophage; 2.5×10^4^ macrophages/mL equivalent to 4 µM of Dox), Lipo-Dox (1.16 µl/mL, equivalent to 4 µM of Dox) or 4 µM of DoxHCl were added and incubated for 2 h, 4 h or 24 h at 37 °C. Dox was used as a fluorescent marker. After incubation, cells were washed three times with PBS, trypsinized, and then analyzed *via* flow cytometry. Pharmacological inhibition of Dox transfer was performed by treating cells with GW4869 (20 μM) for 24 h, CytoB (350 nM) for 6 h, or M-Sec siRNA (50 nM) for 24 h followed by the addition of drugs for 2 h and analysis by flow cytometry 24 h prior to each experiment, 1 ×10^5^ cells/well were plated onto 12 mm borosilicate glass coverslips. LM-Dox, M-Dox or Lipo-Dox (equivalent to 4 μM of Dox) Drugs were added and incubated for 0.5 h, 2 h or 8 h at 37°C. After incubation, the cells were washed three times with PBS, fixed in PFA, stained with Hoechst dye (33342, Thermo Scientific) for 15 min, washed three times, and mounted on glass slides using Prolong Gold antifade reagent (Molecular Probes). Cells were visualized by the laser scanning microscope. Image acquisition was performed by Olympus fluoview3000 software (FV31S-SW).

### Tumor tropism of LM-Dox *in vivo*

Human orthotopic non-small cell lung cancer xenograft model was established by intrapleural injection in the BALB/c nude mice. In brief, 5 × 10^6^ A549/GFP cells were dispersed in a solution containing 25 μL of culture medium and 25 μL of mouse sarcoma extracellular matrix (Matrigel, BD Biosciences, NJ, USA). Cell suspension was quickly injected through the intercostal space into the right lung to a depth of 5 mm using a 29 G needle permanently attached to a 0.5 mL insulin syringe (Becton Dickinson, NJ, USA). After tumor cells injection, the mouse was turned to the right lateral decubitus position. Animals were observed for 30 min until fully recovered.

After three weeks, tumor-bearing mice were intravenously injected with DiD-labeled LM (LM-DiD) and DiD-labeled lipos (Lipo-DiD), respectively. The mice were killed 24 h after injection. Major organs (hearts, lungs, livers, spleens and kidneys) were collected for fluorescence imaging. Fluorescence imaging was performed using the Night OWLIILB983 (Berthold Technologies). All images were acquired with an exposure time of 5 s. The photographic and fluorescent images were individually acquired and then overlaid.

### *In vivo* therapeutic efficacy

The tumor-bearing mice were randomly assigned to 9 groups. LM-Dox, M-Dox, LM+Dox, M+Dox, Lipo-Dox and DoxHCl with the equivalent Dox concentration of 2 mg/kg were administered intravenously every 3 days for 3 weeks. Another three groups were given LM or inactive macrophage vehicles with the equivalent cell amounts of LM-Dox, and vehicle solution 5% glucose, respectively, as the control groups. At day 28, 3 days after the last administration, mice were sacrificed and organs were harvested, photographed and weighed. Then, major organs were subjected to histopathological examination after being fixed in 10% neutral formalin and desiccated and embedded in paraffin.

After the treatment period, blood was collected at day 28. Blood samples were immediately centrifuged at 3,000 rpm for 20 min to harvest serum, which were used to detect IL-10 and TNF-α concentrations with the corresponding assay kits. Serum biochemical parameters of liver function were analyzed by Automatic Biochemical Analyzer (HITACHI, Japan). Liver function indexes include serum alanine aminotransferase (ALT), aspartate aminotransferase (AST), alkaline phosphatase (ALP), albumin (ALB) and total bilirubin (TBIL).

### Statistical analysis

All statistical analysis was performed using Prism 7.0 software (GraphPad Software) by an unpaired Student's t-test, one-way or two-way ANOVA with Bonferroni multiple comparisons post-test. Statistical significance for survival curve was calculated by the log-rank test. Data were approximately normally distributed and variance was similar between the groups. Statistical significance is indicated as * P < 0.05, ** P < 0.01, *** P < 0.001, and **** P < 0.0001.

## Results and discussion

### LM-Dox exhibit tumor tropism in response to CCL2 in an orthotopic lung cancer model

**Figure 1:**
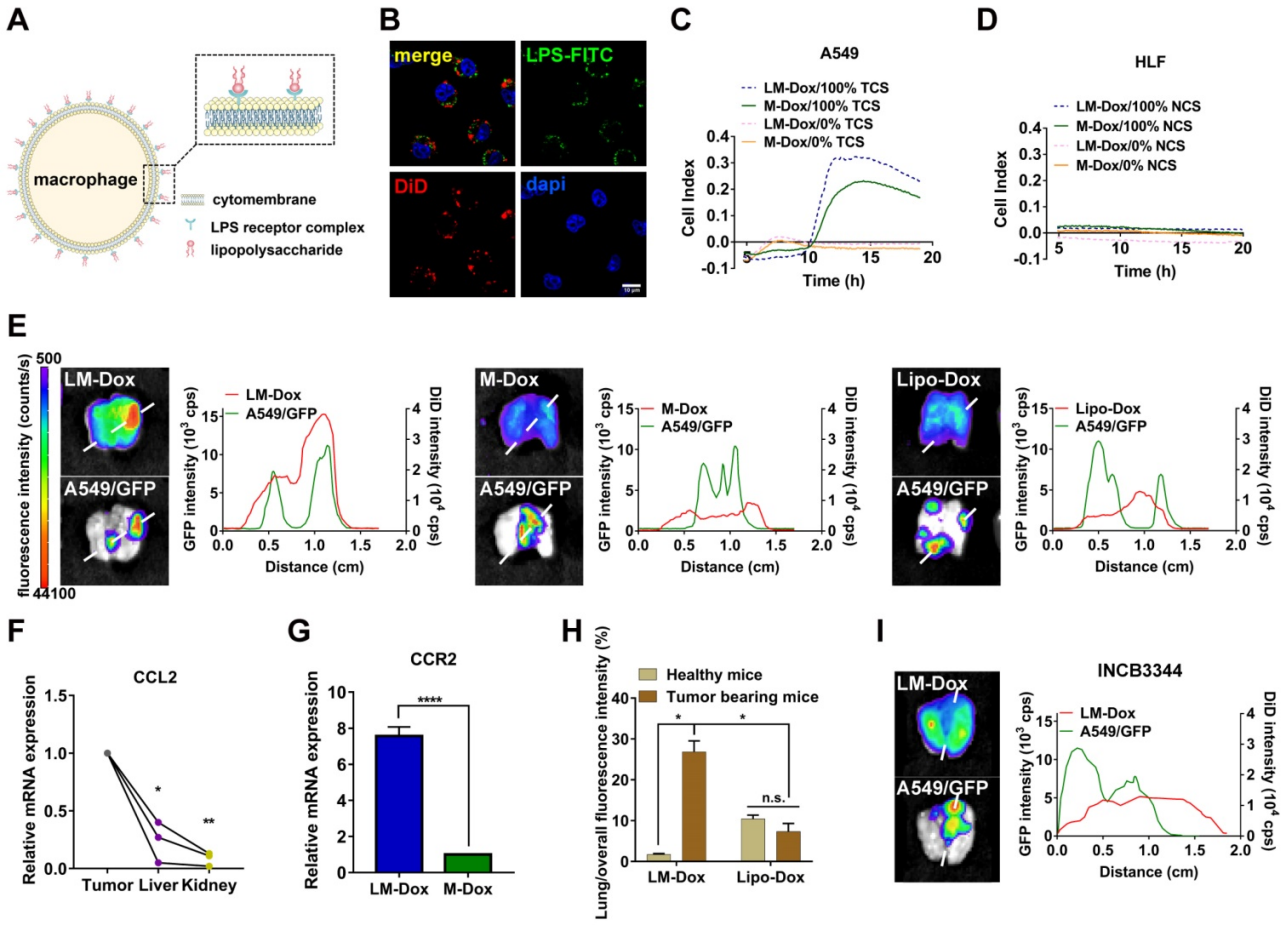
Evaluation of tumor tropism of LM-Dox *in vitro* and *in vivo*. (A) Schematic diagram showing the binding of LPS to macrophages. (B) CLSM images showed LPS-FITC anchored on the surface of macrophages at 48 h. The nuclei were stained with DAPI and plasma membranes were labeled with DiD. LPS-FITC produced a green fluorescence. The merged images were the overlay of three individual images. Scale bar, 10 μm. (C-D) Representative real-time traces of LM-Dox and M-Dox towards human lung tumor A549 cell culture supernatants (NCS) and human lung normal HLF cell culture supernatants (TCS). (E) Representative fluorescence images of excised lungs at 24 h post-injection of DiD-labeled LM-Dox, DiD-labeled M-Dox and DiD-labeled Lipo-Dox (left). The fluorescence intensity of DiD signal and A549/GFP across the lung tissue with cancer (right). (F) Validation of CCL2 gene expression in tumor nodules, liver and kidney by real-time qPCR analysis. (G) Expression of RNA for CCR2 in LM-Dox and M-Dox. Data are derived from quantitative PCR. (H) Lung accumulation of DiD-labeled LM-Dox and DiD-labeled Lipo-Dox in healthy mice and orthotopic lung tumor-bearing mice were calculated by the ratios of DiD fluorescence intensity of lung tissues to total DiD fluorescence intensity. (I) The CCR2 antagonist INCB3344 was administered to mice by oral gavage. 12 hours later, mice were injected with DiD-labeled LM-Dox. Representative fluorescence images of excised lungs at 24 h post-injection of LM-Dox (left). The fluorescence intensity of DiD signal and A549/GFP across the lung tissue with cancer (right). The data are shown as mean ± s.d., * is p < 0.05, ** p < 0.01 by one-way ANOVA test or two-way ANOVA test.

We designed LPS membrane-modified RAW 264.7 macrophages as biologically active carriers for delivery of an anticancer drug Dox, as shown in Figure 1A. CLSM images confirmed that FITC-labeled LPS was anchored on the plasma membrane of macrophages (Figure 1B). Initially, we evaluated the tumor chemotactic responsiveness of LM-Dox by real-time cell migration assays and Dox-loaded macrophages without LPS modification (M-Dox) were used as a control (Figure S1A). Supernatants from human lung cancer A549 cells (TCS) and from normal human lung fibroblast HLF cells (NCS) were used to mimic the tumor microenvironment and normal physiological conditions, respectively. The migration profiles revealed that LM-Dox and M-Dox exhibited high mobility in response to tumor cell culture supernatants; however, there is no migration in the presence of normal cell culture supernatant (Figure 1C, D and S1B, C). Then, we evaluated the *in vivo* tumor accumulation of LM-Dox, which is a prerequisite of precision drug delivery. An orthotopic murine model of lung cancer was established by intrapleural injection of A549/GFP cells in nude mice. The subsequent progression of the lung tumor was monitored using a Night OWLIILB983 imaging systems in live animals. Twenty-four hours after the injection, tumor accumulation of DiD-labeled LM-Dox was significantly higher than that of commercial Lipo-Dox (~ 4-fold) (Figure 1E). To further validate the net tumor accumulation in the lung, we detected fluorescence colocalization of GFP+ lung tumor cells and near-infrared DiD-labeled samples. The results demonstrated that the signals of LM-Dox were completely colocalized with GFP+ tumor cells in the lung tissue, while fluorescence of Lipo-Dox did not localize in close proximity to GFP+ lung tumor cells as shown in Figure 1E. Furthermore, macrophages can be recruited to the tumor sites by CCL2 22. The quantitative PCR and ELISA data validated that mRNA expression of CCL2, which has been shown to mediate macrophage chemotaxis, is significantly upregulated in lung tumor cells and tumor nodules compared with HLF normal cells and normal tissues, respectively (Figure 1F and Figure S1D-E). CCR2 gene expression in response to CCL2 was upregulated by more than 7-fold in LM-Dox (Figure 1G). We blocked the CCL2-CCR2 axis by treatment with INCB3344, a selective rodent-active CCR2 antagonist 23, in animal models to assess the contribution of LM-Dox overexpressing CCR2 to tumor accumulation of the cells. The results demonstrated that pharmacological blockade of the CCL2/CCR2 signaling substantially reduced tumor cell and tissue distribution of LM-Dox in the mouse lung (Figure 1H and Figure S2). Thus, superiority of LM-Dox in tumor accumulation is likely due to recruitment of LPS-anchored macrophages (LM) *via* the CCL2-CCR2 signaling pathway.

To rule out a possibility that high tumor accumulation of LM-Dox results from interception of microsized LM in the lung, intrapulmonary accumulation of LM-Dox in the tumor-bearing mice was compared with that in healthy mice. The results demonstrate that accumulation of LM-Dox in the lung tissue of tumor-bearing mice is increased by 14-fold compared with that in healthy mice treated with the same dose (Figure 1I and Figure S3A). In contrast, intrapulmonary accumulation of Lipo-Dox was very low in healthy mice and in tumor-bearing animals (Figure S3B). LM-Dox accumulate in the lung tumor more efficiently than Lipo-Dox thus providing the basis for cell-to-cell precise drug delivery using LM-Dox.

### LM-Dox hijack tumor microtube networks for precise drug transfer

**Figure 2:**
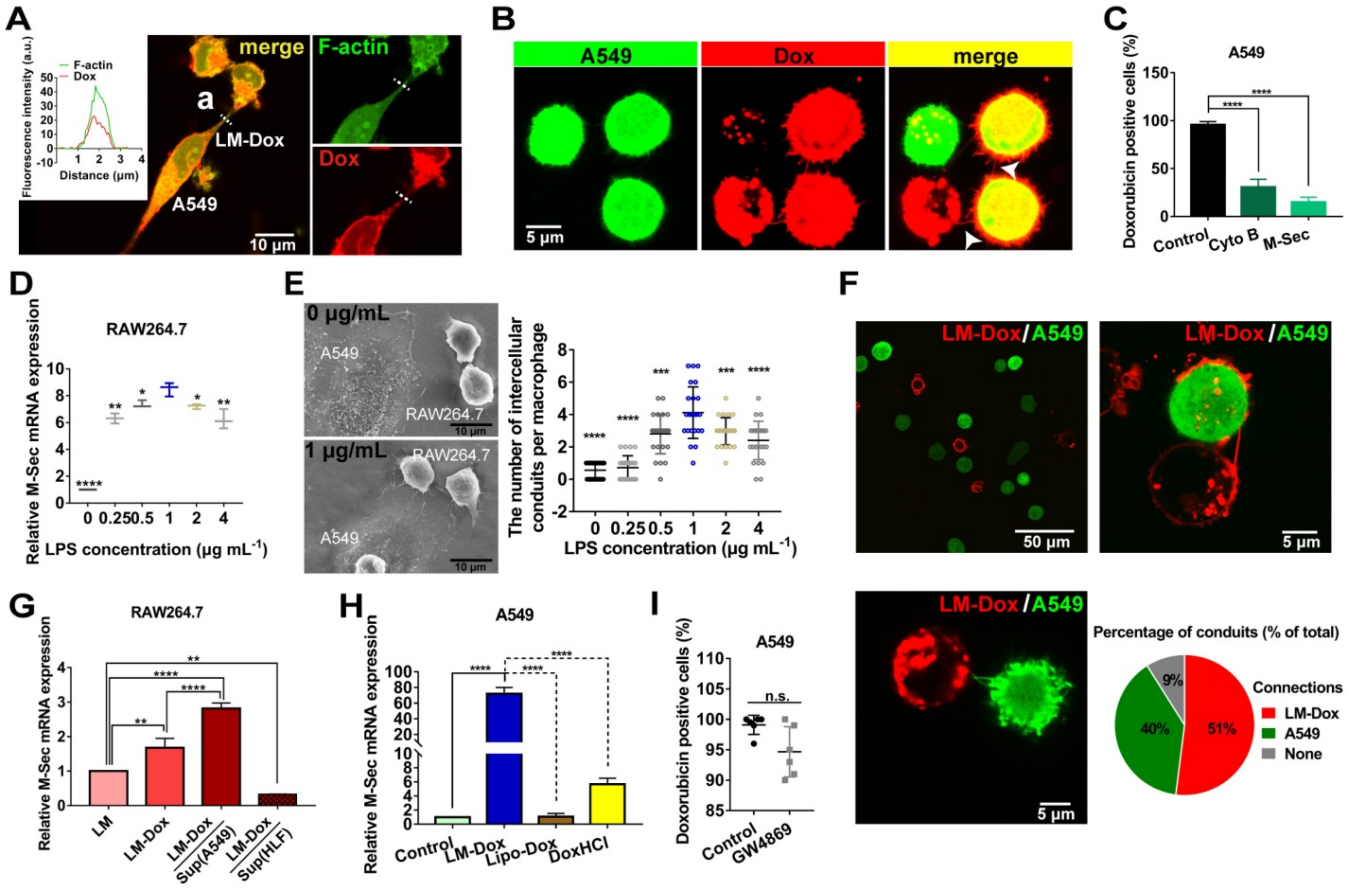
LM-Dox stimulated intercellular conduit formation and hijacked tumor microtube networks of tumor cells for enhancement of drug transfer. (A) Transport of Dox between intercellular conduit-connected LM-Dox and A549 cells. F-actin, the vital component of these membrane conduits was stained with phalloidin (green), and Dox emitted red intrinsic fluorescence. (a) Plot profile of the representative images of fluorescence co-localization of the intercellular conduit and Dox. (B) Images of Dox delivery from LM-Dox to A549 cells *via* intercellular conduits. Intercellular conduits are indicated by white arrows. Scale bar, 5 μm. (C) The Dox positive A549 cells were analyzed by flow cytometry. The A549 cells were pretreated with CytoB and M-Sec siRNA before incubated with LM-Dox (n = 3). (D) Relative quantification of M-Sec mRNA expression in RAW264.7 using real-time qPCR. (E) Quantification of the number of intercellular conduits extended from per macrophage and relevant SEM images of macrophages with LPS modification. Scale bars, 10 µm. (F) Representative CLSM images of intercellular conduits between LM-Dox and A549 cells and the percentage of intercellular conduits protruded from LM-Dox and A549 cells (n = 50). (G) Relative quantification of M-Sec mRNA expression in LM, LM-Dox and LM-Dox stimulated by the supernatants of A549 or HLF cell culture. (H) Real-time qPCR assay for M-Sec mRNA detection of A549 cells with the treatment of LM-Dox, Lipo-Dox and DoxHCl. A549 cells were incubated with LM-Dox, M-Dox, Lipo-Dox and DoxHCl (equivalent to 4 μM of Dox) prior to further measurements. (I) The Dox positive A549 cells were analyzed by flow cytometry. The A549 cells were pretreated with GW4869 before incubated with LM-Dox (n = 6). The data are shown as mean ± s.d., * is p < 0.05, ** is p < 0.01, *** is p < 0.001, **** is p < 0.0001, by one-way ANOVA test.

After accumulation of LM-Dox in the lung, we investigated the means to improve drug transfer efficiency of LM-Dox to the tumor cells. The membrane-based intercellular conduits can transport cell cargo, such as mitochondria, viruses, and miRNAs, between specific cells for intercellular communication 16, 24. Here, confocal imaging documented that LM-Dox can delivered Dox to A549 cells *via* the intercellular conduits between the cells (Figure 2A).

After LM-Dox connects to an A549 tumor cell using the intercellular conduits, LM-Dox can transfer Dox (red) to two A549 cells (green) which have been in contact with each other *via* tumor microtubes. The overlay of Dox red fluorescence over the A549 green fluorescence results in yellow signal of the cells (Figure 2B). However, a significantly lower intensity of Dox fluorescence was observed in an A549 cell that did not form any tumor microtubes with other tumor cells or LM-Dox. Moreover, blockade of the tumor microtubes between A549 cells using a pharmacological inhibitor cytochalasin B (CytoB) or by a gene-silencing agent M-Sec siRNA results in a sharp decrease in Dox transfer efficiency from nearly 100% to less than 30.7% (Figure 2C). It should be noted that LPS acts as a membrane anchor of macrophage to induce M-Sec mRNA expression (Figure 2D), which plays a key role in the formation of intercellular conduits 25 and stimulates intercellular conduit formation by macrophages. Electron microscopy images confirmed that LPS increased the number of intercellular conduits generated by the macrophages (Figure 2E and S4A). In the subsequent experiments, 1.0 μg/ml of LPS was selected as the optimal concentration for macrophage modification and intercellular conduit stimulation. To investigate whether these intercellular conduits originate from LM-Dox or A549 cells, we cocultured the mEGF-lifeact-7 plasmid-transfected A549 tumor cells and DiD-labeled LM and analyzed the intercellular conduit formation between these cells. Most of DiD-labeled LM (91%) are able to connect with A549 tumor cells *via* intercellular conduits. Red fluorescence intercellular conduits (51%) protrude from LM and 40% of green fluorescence intercellular conduits are produced by A549 cells suggesting that LM and A549 tumor cells tend to create intercellular conduits with each other (Figure 2F). Intriguingly, the supernatants from A549 tumor cells and loading with Dox clearly increase M-Sec mRNA expression levels of LM-Dox, whereas supernatants from HLF normal cells inhibit M-Sec mRNA expression (Figure 2G). On the other hand, LM-Dox induces a significant upregulation of M-Sec mRNA expression in A549 tumor cells while Lipo-Dox does not upregulate the expression (Figure 2H). Moreover, exocytosis leads to the extracellular release of exosomes, which are involved in intercellular communication 26; thus, we evaluated the effects of inhibition of exosome secretion 27 on Dox transfer. The results indicate that pretreatment with GW4869 (20 µM) of LM-Dox does not reduce the drug transfer efficiency of LM-Dox (Figure 2I) suggesting that drug transport of LM-Dox is essentially independent from exocytosis.

To examine whether cell-to-cell drug delivery results in higher efficacy, LM-Dox with the optimal Dox loading content of 0.25 pmol/macrophage and the encapsulation efficiency of 83.37% were exposed to lung cancer cell line A549. Drug transfer kinetics analysis demonstrates that the drug transfer rate of LM-Dox and M-Dox was remarkably faster than that in the case of Lipo-Dox (Figure S4B-E). Surprisingly, the percentage of Dox-positive A549 cells was nearly 100% after 0.5 h incubation, which was faster than loading with free DoxHCl that took approximately 8 h of treatment to diffuse into all A549 cells. Rapid drug transfer of LM-Dox led to higher cytotoxicity against A549 tumor cells compared with that of either Lipo-Dox or DoxHCl (Figure S5A-C). Thus, these data indicate that LM-Dox can hijack the established tumor microtube networks and even stimulate the intercellular conduit formation between LM-Dox and A549 cells, but not with HLF normal cells, for rapid delivery of anticancer drugs and subsequent induction of robust apoptosis of A549 cells.

### LM-Dox inhibit the growth of primary lung cancer with a synergistic therapeutic effect of TNF-α release

**Figure 3:**
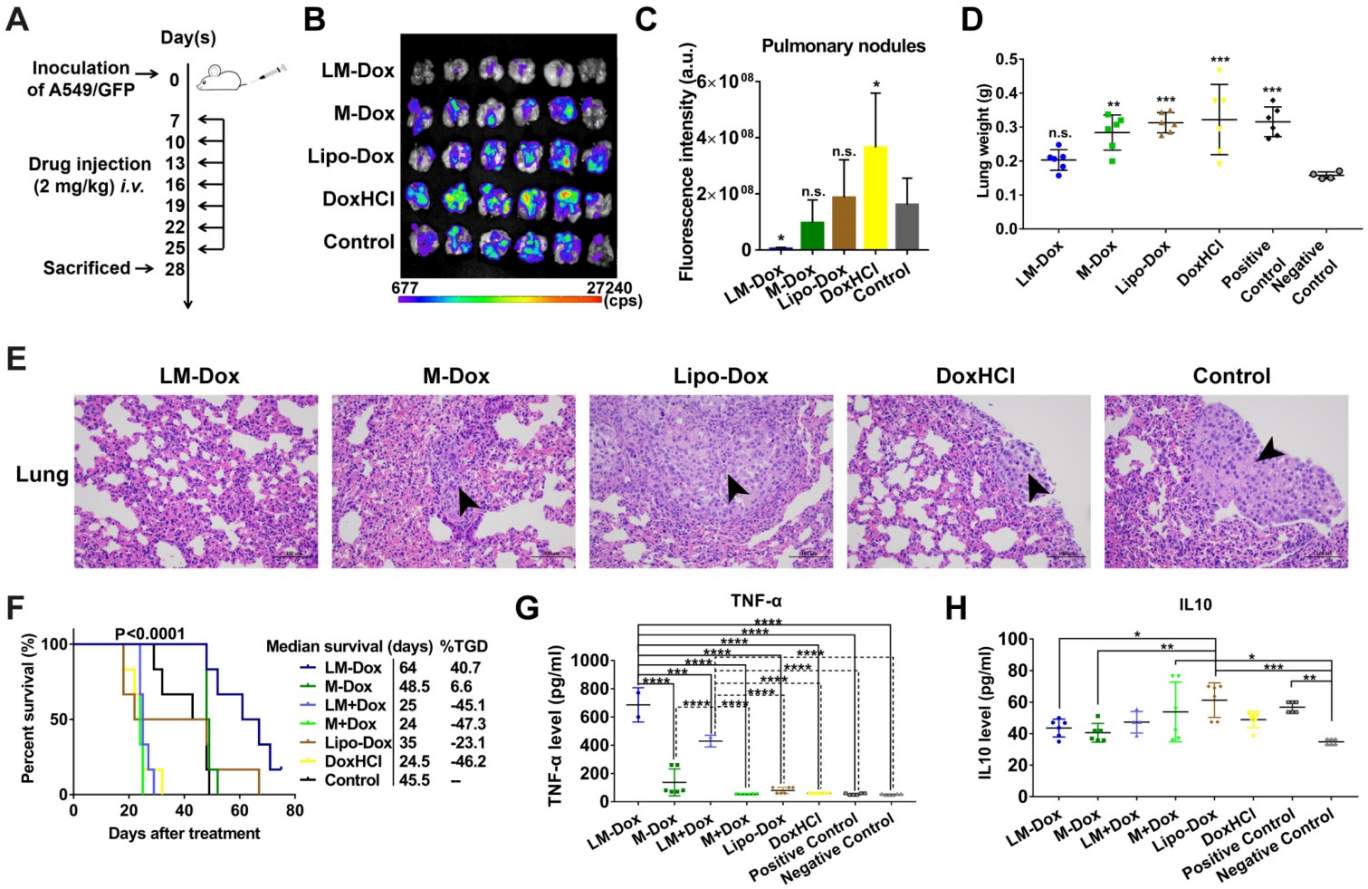
*In vivo* therapeutic efficacy of LM-Dox against orthotopic lung cancer. (A) Experimental procedure of tumor induction and treatment protocol. (B) Fluorescence imaging of lungs harvested from treated and control mice on day 28. Fluorescence indicated the location of the GFP+ tumors. (C) Corresponding quantitative fluorescence intensity of lungs (n = 6). (D) Organ weights of lung after 28 days of treatment (n = 6). (E) H&E stained lungs from control and treated tumor-bearing mice on day 28. Black arrows indicate tumor cells. Scales are shown below each image. (F) Kaplan-Meier survival curves and the median survival time of lung tumor-bearing mice after different treatments. (n = 6). Treated group (T) - Control group (C) = difference between median survival(days) of T *vs.* C (TGD). (T - C)/C (%TGD). Serum was isolated from whole blood of the experimental mice. Levels of (G) TNF-α and (H) IL-10 were measured by ELISA. The data are shown as mean ± s.d., * p < 0.05, ** p < 0.01, *** p < 0.001, **** p < 0.0001, n.s. is p > 0.05 by one-way ANOVA test.

We evaluated the potential therapeutic application of LM-Dox in a mouse orthotopic lung cancer model as shown in Figure 3A. The antitumor effects were evaluated by measuring the relative fluorescence intensity of the GFP-expressing tumor cells. Dosing of the animals with 2 mg of either DoxHCl or Lipo-Dox had no effect on primary tumor growth compared with the untreated tumor-bearing control mice. However, the fluorescence intensity of GFP+ tumor cells in the lungs of mice treated with LM-Dox was decreased by approximately 35-fold compared with that in the control mice suggesting that LM-Dox produces a robust inhibition of the primary lung tumor growth (97% inhibition *vs.* control) (Figure 3B and 3C). Moreover, after LM-Dox treatment, the lung weight was comparable to that of the healthy mice. In the other treatment groups (M-Dox, Lipo-Dox and DoxHCl), a significant lung weight increase was observed due to tumor growth (Figure 3D); these data were confirmed by the results of the hematoxylin and eosin (H&E) staining (Figure 3E). Impressively, the mice treated with LM-Dox survived over the entire 64-d duration of the experiment and had significantly improved median survival time by 161.2% and 82.9% compared with that in the DoxHCl and Lipo-Dox treatment groups, respectively (Figure 3F).

Blood serum samples were collected for hematological analysis. Interestingly, a significant increase in the levels of TNF-α, which is cytotoxic to a wide variety of tumor cells and inhibits tumor growth 28, was detected in the LM-Dox-treated group (Figure 3G). Similarly, upregulated levels of TNF-α were detected after separate administration of empty LM in combination with free Dox. On the other hand, this result is consistent with the data of the literature that described an increase in the serum levels of interleukin 10 (IL10), which is an immunosuppressive factor that suppresses production of TNF-α 29, in untreated tumor-bearing mice, whereas LM-Dox treatment was able to overcome the upregulation of IL10 (Figure 3H). These findings prompted us to investigate whether LM-Dox can induce TNF-α release to increase the cytotoxicity against tumor cells.

### LM-Dox activate tumor-associated macrophages to express TNF-α

**Figure 4:**
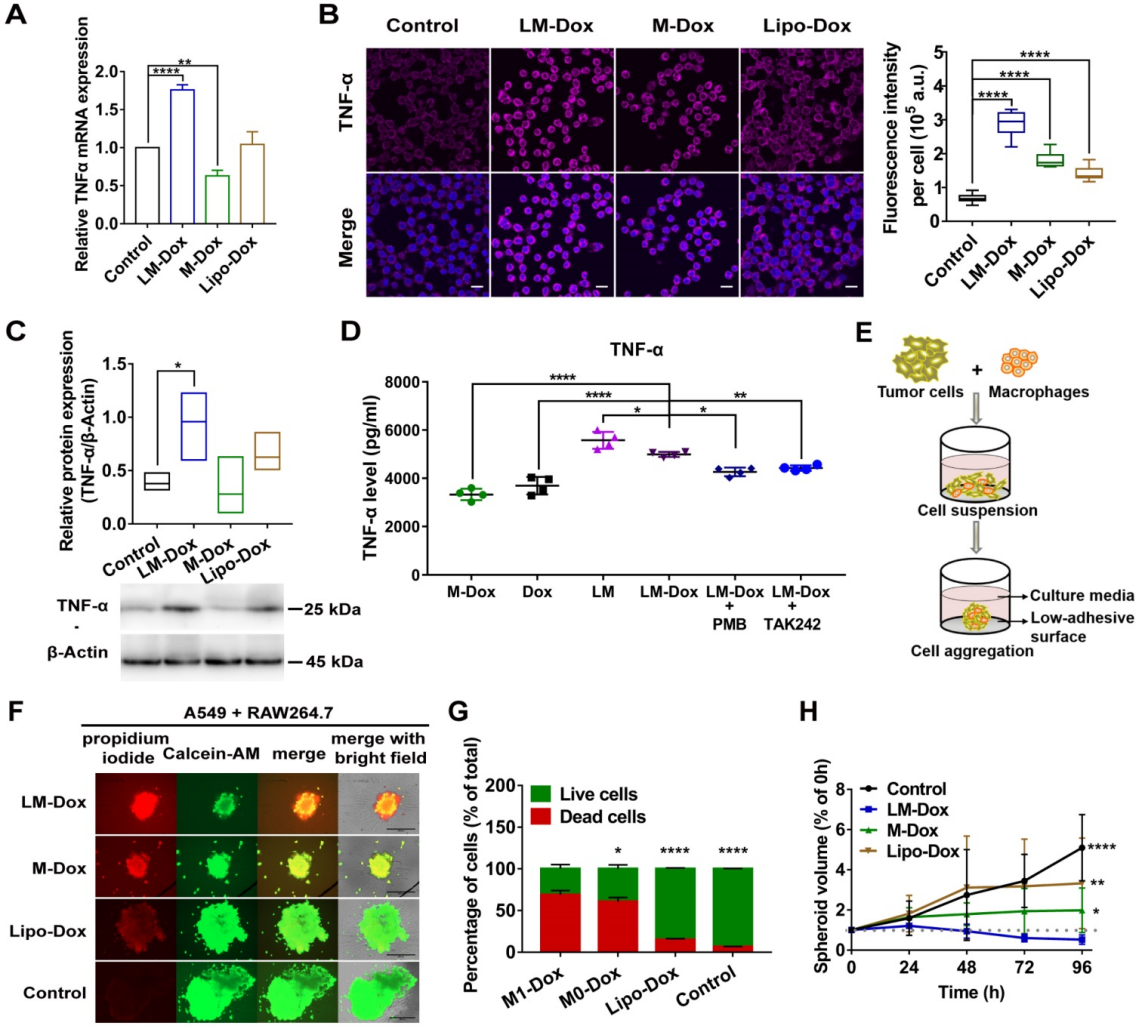
LM-Dox induced TNF-α secretion from TAMs. (A) Total RNA isolated from TAMs after exposure to LM-Dox, M-Dox and Lipo-Dox was examined by real-time qPCR and the mRNA levels of TNF-α were normalized to the control GAPDH mRNA levels. (B) Confocal microscopic images of immunofluorescent labeling for TNF-α (purple) in TAMs. Cell nuclei were stained with DAPI (blue) in all images. Scale bars, 20 µm. Quantitative analysis of fluorescent intensity was performed in at least ten independent visual fields of immunofluorescence images. (C) Quantification of protein levels of TNF-α in TAMs. Whole-cell extract was prepared and quantified. An equivalent amount of total protein was analyzed with antibodies against TNF-α and β-Actin as the loading control. (D) The TNF-α levels in the culture medium were analyzed by ELISA. PMB was used to block the effect of LPS-induced activity. TAMs were pretreated with TAK242 before incubated (n = 3). (E) Schematic depicting tumor-immune spheroid formation where cell spheroids have been generated by culturing A549 tumor cells in combination with RAW264.7 macrophages. (F) Calcein-AM (green)/PI (red) assay was used to visualize the live cells (green) and dead cells (red) in spheroids. Scale bars, 400 μm. The spheroids were incubated with LM-Dox, M-Dox and Lipo-Dox, respectively (equivalent to 4.0 µM Dox) for 72 h. (G) The relative fluorescence intensities of the red-fluorescent (PI, λ_ex_ 535 nm, λ_em_ 617 nm) dead cells versus the green-fluorescent (Calcein-AM, λ_ex_ 490 nm, λ_em_ 515 nm) live cells were determined, indicating a significant increase in cell death followed by the LM-Dox treatment. (H) The volume of spheroids was calculated and normalized to the volume before treatments. Statistical analysis of images was based on ImageJ quantification of randomly selected fields of spheroids (n > 5) for each treatment. The data are shown as mean ± s.d., * is p < 0.05, ** is p < 0.01, *** is p < 0.001, **** is p < 0.0001, n.s. is p > 0.05 by one-way ANOVA test.

Tumor-associated macrophages (TAMs) are the major stromal component within the tumor; hence, we aimed to determine whether LM-Dox can induce TNF-α release by TAMs. Initially, we prepared TAMs by culturing RAW264.7 macrophages with A549 tumor cell-conditioned medium for 2 days. Western blot analysis identified TAMs by an increase in the expression of CD206 and arginase-1 (Figure S6A). After treatment of TAMs with LM-Dox, expression levels of TNF-α mRNA were substantially elevated compared with that in untreated TAMs; M-Dox or Lipo-Dox did not elevate the expression levels (Figure 4A and Figure S6B). Immunofluorescence, western blot and ELISA analysis confirmed that LM-Dox treatment induces elevated TNF-α expression in TAMs that released significant amount of bioactive TNF-α in the supernatant (Figure 4B and C). LPS can induce TNF-α gene expression 30; thus, we performed loss-of-function studies to validate membrane-anchored LPS induction of TNF-α release. An inhibitor of LPS biological activity, PMB, and a TLR4 signaling blocker, TAK242, attenuated TNF-α gene expression in TAMs (Figure 4D and Figure S6C). Thus, these results suggest that LM-Dox activate tumor-associated macrophages to produce TNF-α through membrane-anchored LPS.

Then, we explored whether LM-Dox-induced secretion of TNF-α by TAMs can enhance antitumor efficacy of Dox. Thus, we constructed A549-RAW264.7 (5:1) multicellular spheroids as a 3D tumor-immune model (Figure 4E and Figure S6D). After exposure to LM-Dox or control treatments for 96 h, cell viability was assessed using a fluorescence-based live/dead cell assay kit. Calcein-AM stains live cells with green fluorescence, while propidium iodide stains dead cells with red fluorescence (Figure 4F). LM-Dox induction of cell death in tumor-immune spheroids was more robust compared with that induced by Lipo-Dox treatment under identical conditions (Figure 4G) resulting in a strong inhibition of tumor-immune spheroid growth (Figure 4H and S6E). Hence, LM-Dox induces TNF-α release to increase the cytotoxicity of Dox against A549 tumor cells to support therapeutic potential of LM-Dox *in vivo*.

### LM-Dox inhibit liver metastasis of lung cancer

**Figure 5:**
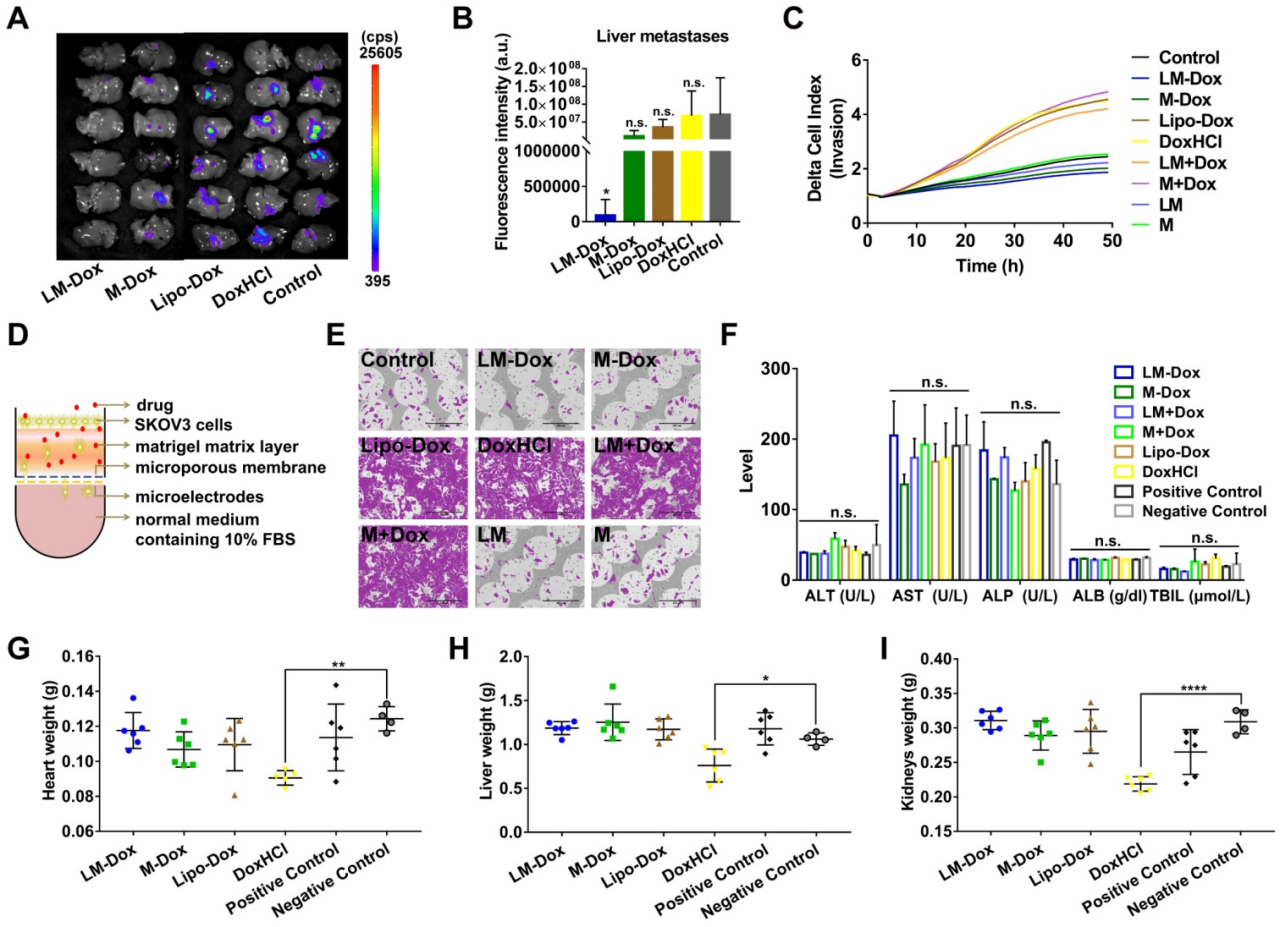
Inhibition of liver metastasis and minimization of the side effects induced by Dox. (A) Fluorescence imaging of livers harvested from treated and control mice on day 28. Fluorescence indicated the location of the GFP+ tumors. (B) Corresponding quantitative fluorescence intensity of livers (n = 6). (C) Representative profiles comparing the invasion rate of A549 cells induced by various samples was monitored by using real-time cell analyzer. (D) Schematic illustration of a real-time invasion assay. (E) SEM images of the microporous membranes in the CIM-16 well plates after finishing the real-time invasion assay in (C). Scale bars, 200 µm. (F) Serum biochemical parameters of liver function were analyzed. Organ weights of (G) heart, (H) liver and (I) kidneys after 28 days of treatment (n = 6 per group). The data are shown as mean ± s.d., * p < 0.05, ** p < 0.01, *** p < 0.001, **** p < 0.0001, n.s. is p > 0.05 by one-way ANOVA test.

Liver is one of the most frequent targets of metastasis of lung cancer 31; hence, we evaluated the effects of LM-Dox on liver metastasis in the orthotopic lung cancer model with GFP-expressing tumors. After 28 days of treatment, the livers were sectioned and metastatic GFP-expressing tumor cells were analyzed. The livers of the LM-Dox treated mice had significantly lower fluorescence intensity than that in the untreated control suggesting effective inhibition of the primary tumor metastasis to the liver (Figure 5A, B). In contrast, commercial Lipo-Dox or DoxHCl did not have any antitumor effects on liver metastasis. Previous studies revealed that chemotherapy can kill tumor cells while also promoting tumor invasion 32. We investigated whether LM-Dox can overcome the Dox-induced tumor invasion and can inhibit liver metastasis. We monitored the rate and onset of A459 cell invasion after exposure to LM-Dox and to control treatments by using an xCELLigence real-time cell analyzer (Figure 5D). The invasion kinetics profiles indicate that Dox-treated A549 tumor cells demonstrate a strong invasive phenotype and that Lipo-Dox and DoxHCl follow the same trend. In contrast to this, an intriguing observation indicates that LM-Dox- and M-Dox-treated A549 tumor cells have low or non-existent migration compared with that in the untreated cells (Figure 5C, E). Collectively, these data imply that LM-Dox is able to remarkably suppress liver metastasis partly due to inhibition of Dox-induced tumor invasion.

In addition to therapeutic efficacy, we evaluated *in vivo* side effects of LM-Dox. After 7 i.v. injections, results of biochemical analysis and organ weight were evaluated. Multiple biochemical analyses showed that the levels of alanine aminotransferase (ALT), aspartate aminotransferase (AST), alkaline phosphatase (ALP), albumin (ALB) and total bilirubin (TBIL) are within the normal range in all groups (Figure 5F). Moreover, injection with LM-Dox did not induce weight loss in the heart, liver and kidneys. However, DoxHCl exhibited a noticeable multi-organ toxicity (Figure 5G-I) which is a typical toxic side effect caused by chemotherapy with free Dox.

## Conclusions

The present study shows that engineering LMs-Dox, an LPS-anchored macrophage-based drug delivery platform, robustly suppresses tumor growth and metastasis and significantly reduces the side effect of Dox compared with liposome-based drug delivery system *in vitro* and *in vivo*. In response to high expression levels of CCL2 in tumor microenvironment, LM-Dox exhibit tumor tropism and subsequently hijack the established tumor microtubes of lung tumor cells to selectively deliver the chemotherapeutic drugs. Simultaneously, LM-Dox induces secretion of TNF-α by tumor-associated macrophages to increase the cytotoxicity of Dox. LM-Dox may serve as versatile bioactive carriers of chemotherapeutic drugs. In the clinical context, these engineered microphages represent a personalized medicine approach that can be translated into the potential use of patient-derived monocytes/macrophages for drug delivery by the means of cell-to-cell communication.

## Supplementary Material

Supplementary figures and tables.

## Abbreviations

TNF-α: tumor necrosis factor alpha
CCL2: monocyte chemoattractant protein-1
CCR2: C-C chemokine receptor type 2
LPS: lipopolysaccharide
TLR4: toll-like receptor 4
TAMs: tumor-associated macrophages
DoxHCl: doxorubicin hydrochloride
PMB: polymyxin B
DAPI: 4′, 6-Diamidino-2-phenylindole dihydrochloride
DMSO: dimethyl sulfoxide
MTT: 3-(4,5-dimethylthiazol-2-yl)-2,5-diphenyl tetrazolium bromide
GFP: green fluorescent protein
ALT: alanine aminotransferase
AST: aspartate aminotransferase
ALP: alkaline phosphatase
ALB: albumin
TBIL: total bilirubin
