# Supplementary material for: Lipopolysaccharide-anchored macrophages hijack tumor microtube networks for selective drug transport and augmentation of antitumor effects in orthotopic lung cancer
Source: Theranostics. 2019 Sep 21;9(23):6936–48. doi: 10.7150/thno.37380 (PMC6815965; doi:10.7150/thno.37380)
Supplement: Supplementary file 1 — Supplementary figures and tables. [file thnov09p6936s1.pdf]

Lipopolysaccharide-anchored macrophages hijack tumor microtube networks for selective drug transport and augmentation of antitumor effects in orthotopic lung cancer

Ling Guo, Ye Zhang, Runxiu Wei, Cuifeng Wang, Min Feng**

School of Pharmaceutical Sciences, Sun Yat-sen University, University Town, Guangzhou, 510006, P.R. China

*Corresponding authors: Tel.: +8620 3994373; fax: +8620 3994373

Email address: wangcf6@mail.sysu.edu.cn and fengmin@mail.sysu.edu.cn

Contents

1. Supplementary methods.. S3

2. Supplemental figures S4

Supplementary methods

Cytotoxicity assay.

A549 cells were incubated for 24 h at 5000 cells/well using 96-well plates (Costar, U.S.A). When reaching to 70% ~ 80% confluence, the cells were exposed to varying concentrations of LM-Dox or M-Dox for another 48 h. To determine cell viabilities, 20 μL of MTT solution (5 mg/mL, in phosphate buffered saline) was added to 100 μL of medium in each well of the 96-well plate. The plate was placed in a cell culture incubator until purple precipitates were clearly visible (approximately 4 h). Then 150 μL of dimethyl sulphoxide (DMSO) was added. The absorbance in each well was measured at 570 nm in a microtiter plate reader (Bio-Tek, U.S.A).

To compare the cytotoxicity of different formulations, A549 cells were exposed to LM-Dox, M-Dox, LM+Dox, M+Dox, Lipo-Dox and DoxHCl (equivalent to 1.148 μM Dox which is the IC_{50} value of LM-Dox against A549 cells for 48 h) for 24 h.

Apoptosis assay.

Annexin-V/PI labeling was performed according to the manufacturer's recommendations. Apoptosis assay was performed on a flow cytometer (EPICS XL, Beckman Coulter, USA). 2.0×10^5 cells were treated with cell culture medium (control), LM-Dox, M-Dox, Lipo-Dox and DoxHCl at the concentration of 1.148 μM for A549 cells. After 48 h incubation, cells were washed with 400 μL PBS twice and resuspended in 400 μL binding buffer. Cells were stained with 10 μL Annexin V-FITC and 5 μL propidium iodide (PI) solution provided in the kit in dark for 15 min. At least 10000 cells were analyzed to determine the percentage of dead cells.

Supplemental figures

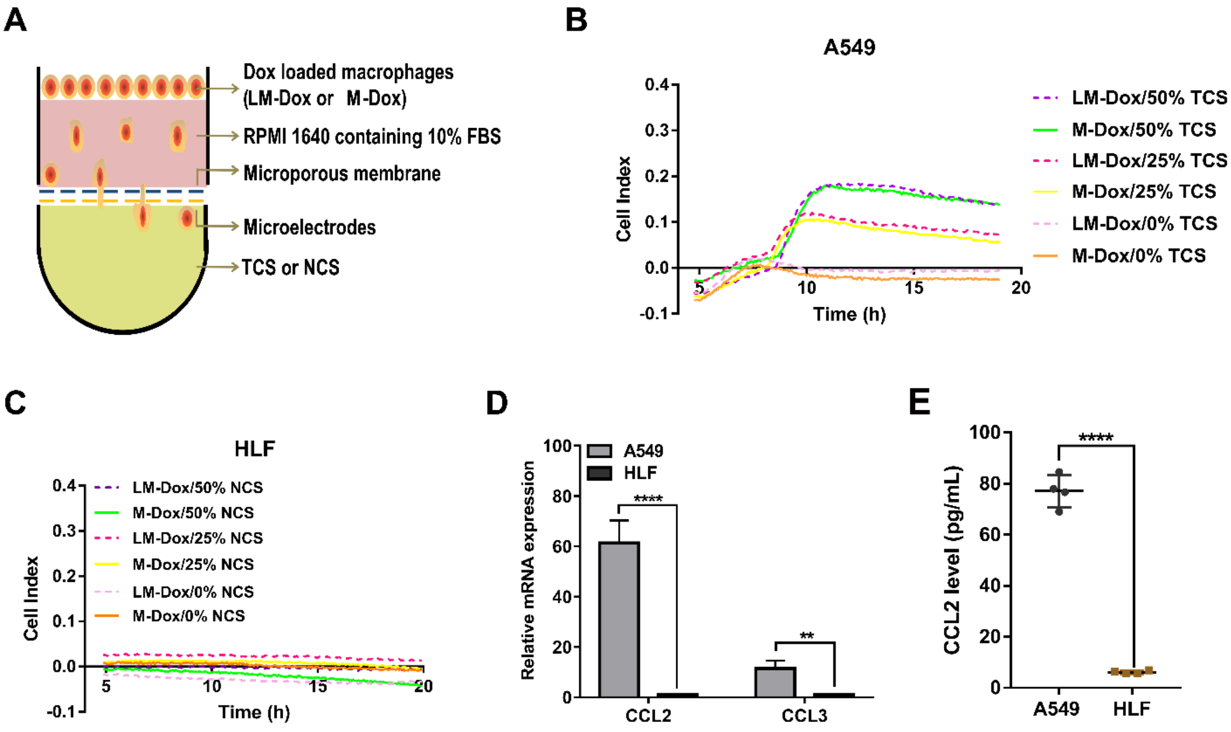

Figure S1. (A) Schematic illustration of a real-time tropism assay. (B-C) Representative real-time traces of LM-Dox and M-Dox towards (B) A549 tumor cell culture supernatants and (C) HLF normal cell culture supernatants (TCS: tumor cell culture supernatants; NCS: normal cell culture supernatants). (D) Quantification of relative CCL2 and CCL3 chemokine expression in A549 cells and HLF cells by real-time qPCR analysis. (E) ELISAs were performed to examine the production of CCL2 in the cell culture supernatants of A549 cells compared with HLF cells. The data are shown as mean ± s.d., ** is $p < 0.01$, **** is $p < 0.0001$ by one-way ANOVA test or two-way ANOVA test.

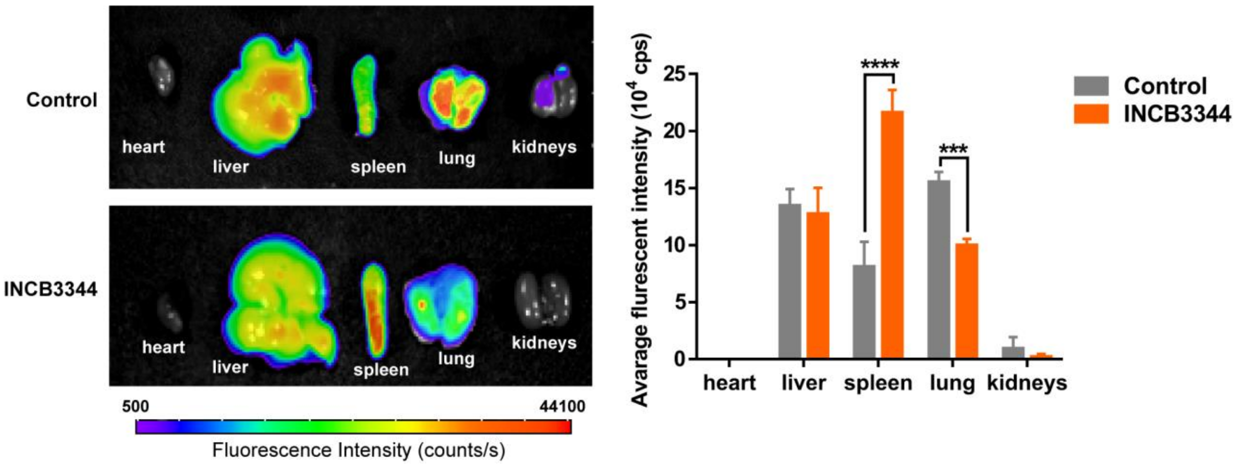

Figure S2. Biodistribution of DiD-labeled LM-Dox at 24 h was measured by fluorescence imaging in the heart, liver, spleen, lung and kidneys. INCB3344 was administered to mice by oral gavage 12 hours before treatment with LM-Dox. The data are shown as mean ± s.d., *** is $p < 0.001$, **** is $p < 0.0001$ by two-way ANOVA test.

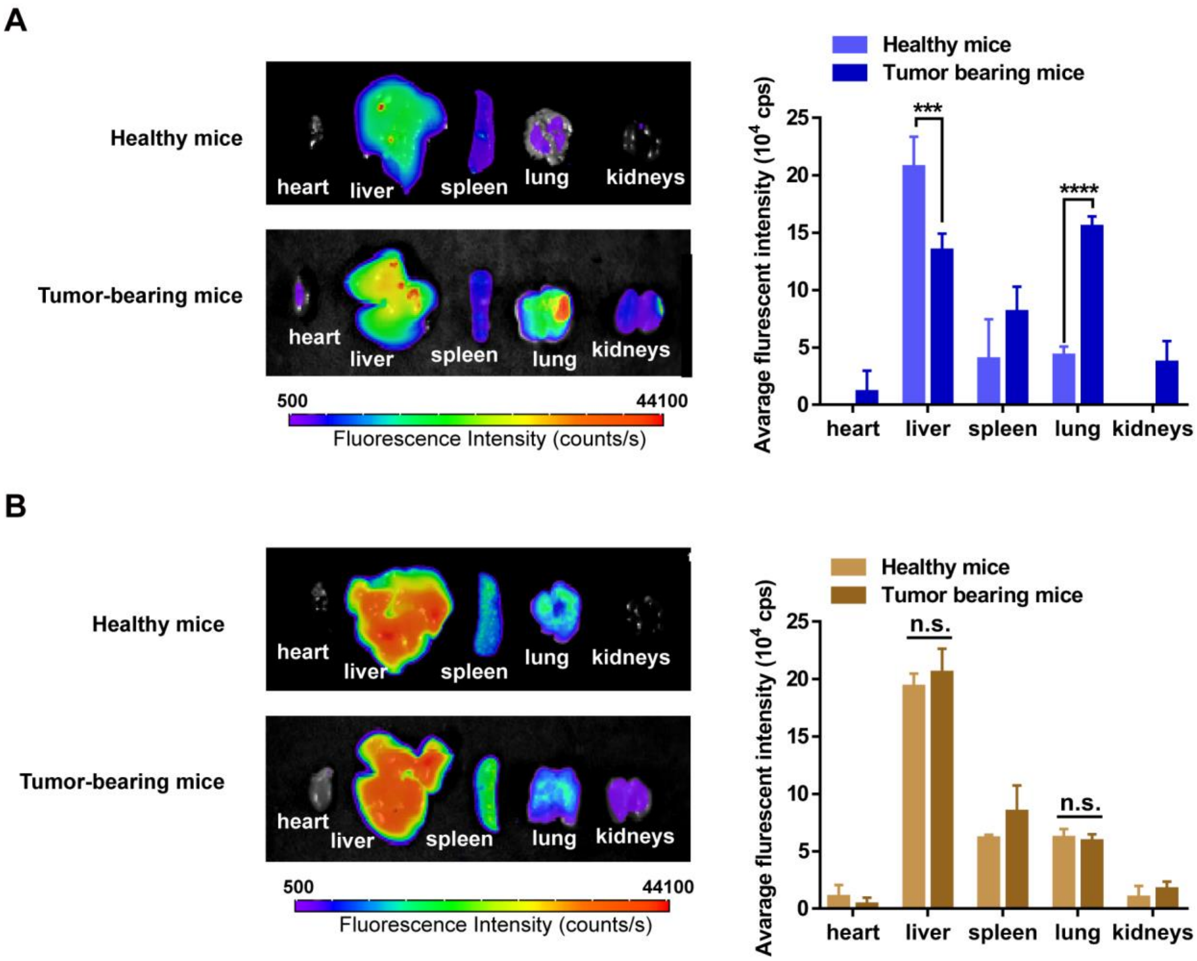

Figure S3. Biodistribution of DiD-labeled LM-Dox (A) and DiD-labeled Lipo-Dox (B) at 24 h was measured by fluorescence imaging in the heart, liver, spleen, lung and kidneys. The data are shown as mean ± s.d., *** is $p < 0.001$, **** is $p < 0.0001$, n.s. is $p > 0.05$ by two-way ANOVA test.

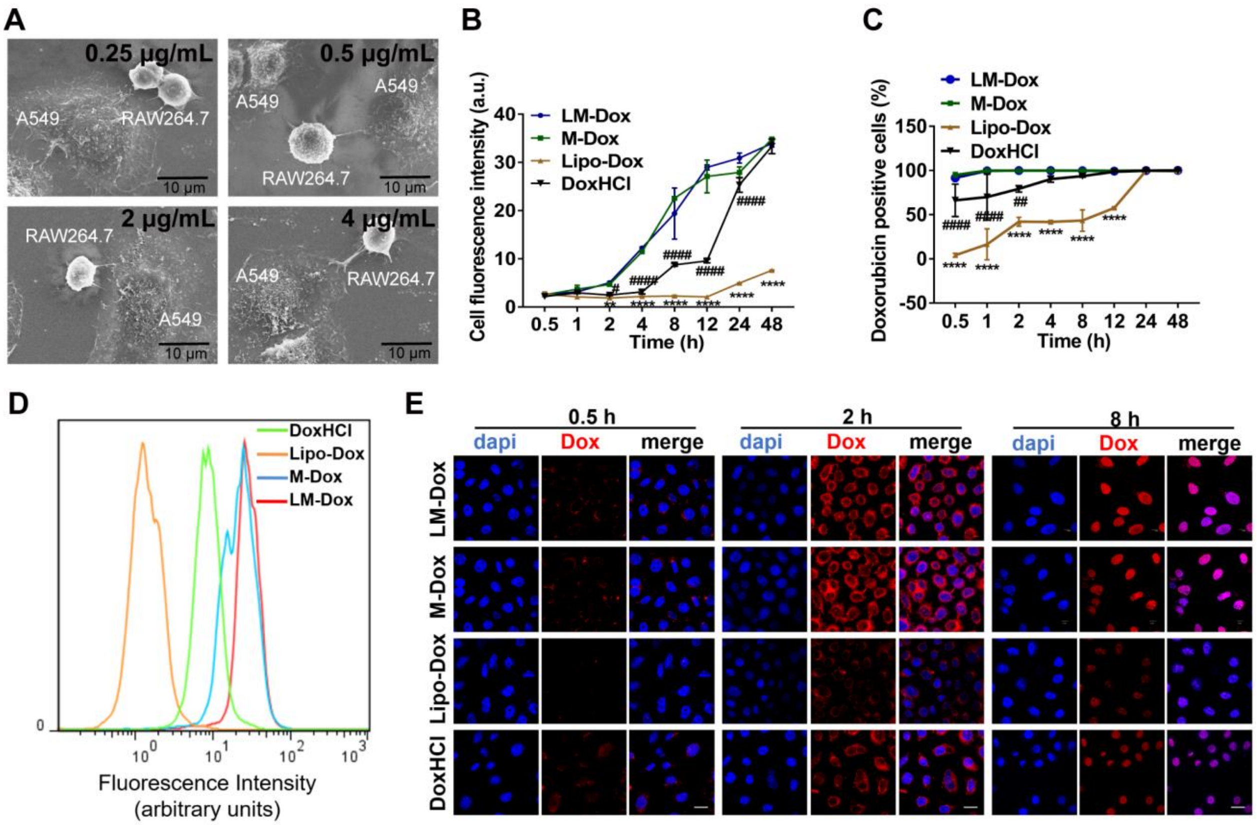

Figure S4. (A) SEM images of macrophages after LPS stimulation. Scale bars, 10 μm. (B-C) Percentages of Dox positive cells and the mean doxorubicin fluorescence intensities of A549 cells incubated with LM-Dox, M-Dox, Lipo-Dox or DoxHCl were measured by flow cytometry at the indicated times. (D) The representative fluorescence intensities of Dox-positive A549 cells at 4h were displayed. (E) CLSM images of A549 cells show the Dox transfer into A549 tumor cells at 0.5 h, 2 h and 8 h. The nuclei were stained with DAPI. Dox produced a red fluorescence. The merged images were the overlay of two individual images. Scale bar, 20 μm.

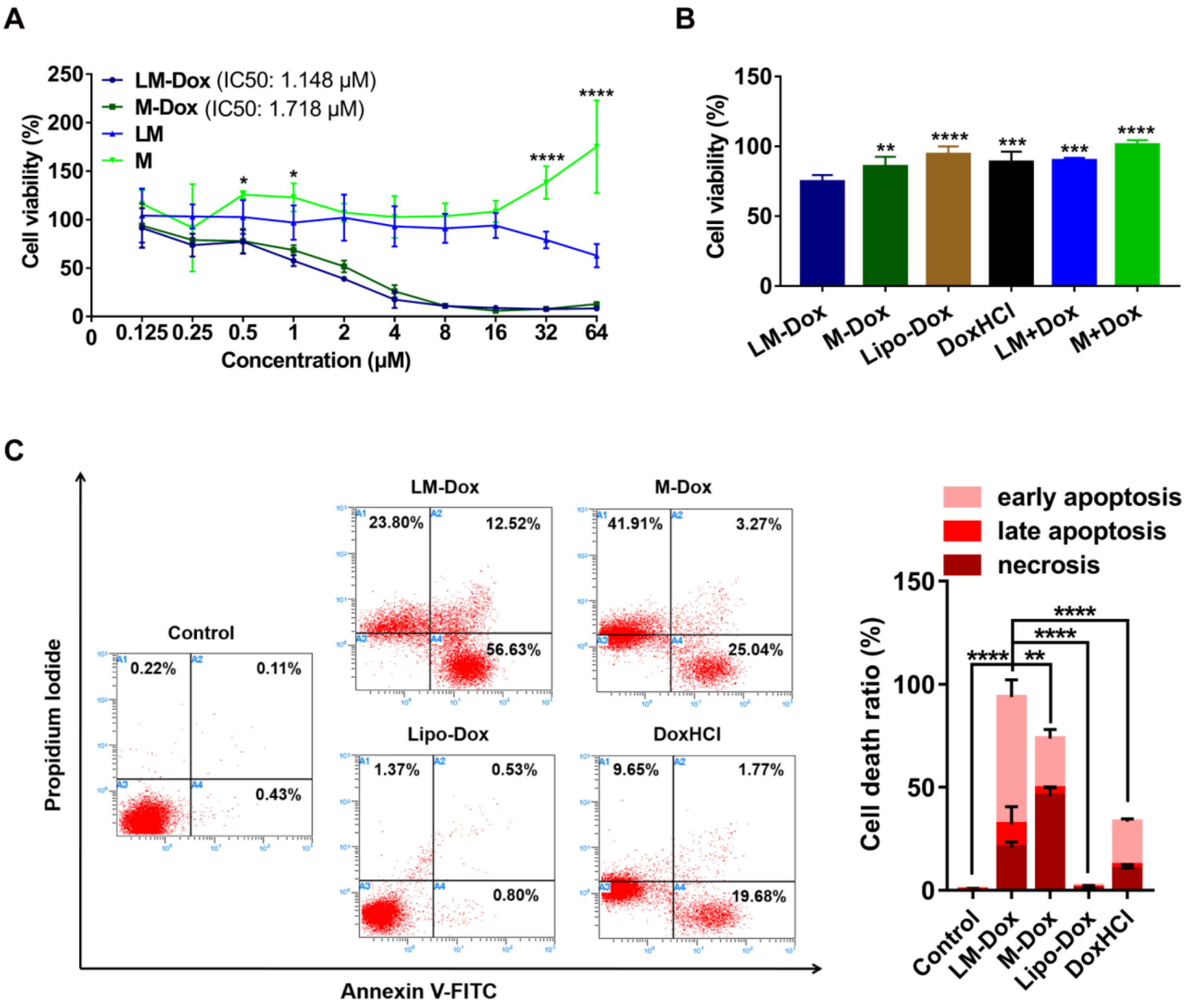

Figure S5. (A) Dose-response curves for cell viability of A549 cells with treatment of LM-Dox, M-Dox, LM and macrophages for 48 h. (B) Cell viability of A549 cells after exposure to various samples for 24 h (equivalent to 1.148 μM Dox). (C) Annexin V-FITC/PI assay for apoptosis detection of A549 cells treated with LM-Dox, M-Dox, Lipo-Dox and DoxHCl (equivalent to 1.148 μM Dox) for 48 h. Quantitative data showed the percentage of early apoptotic, late apoptotic and necrotic cells. The data are shown as mean ± s.d., * is $p < 0.05$, ** is $p < 0.01$, *** is $p < 0.001$, **** is $p < 0.0001$, n.s. is $p > 0.05$ by one-way ANOVA test or two-way ANOVA test.

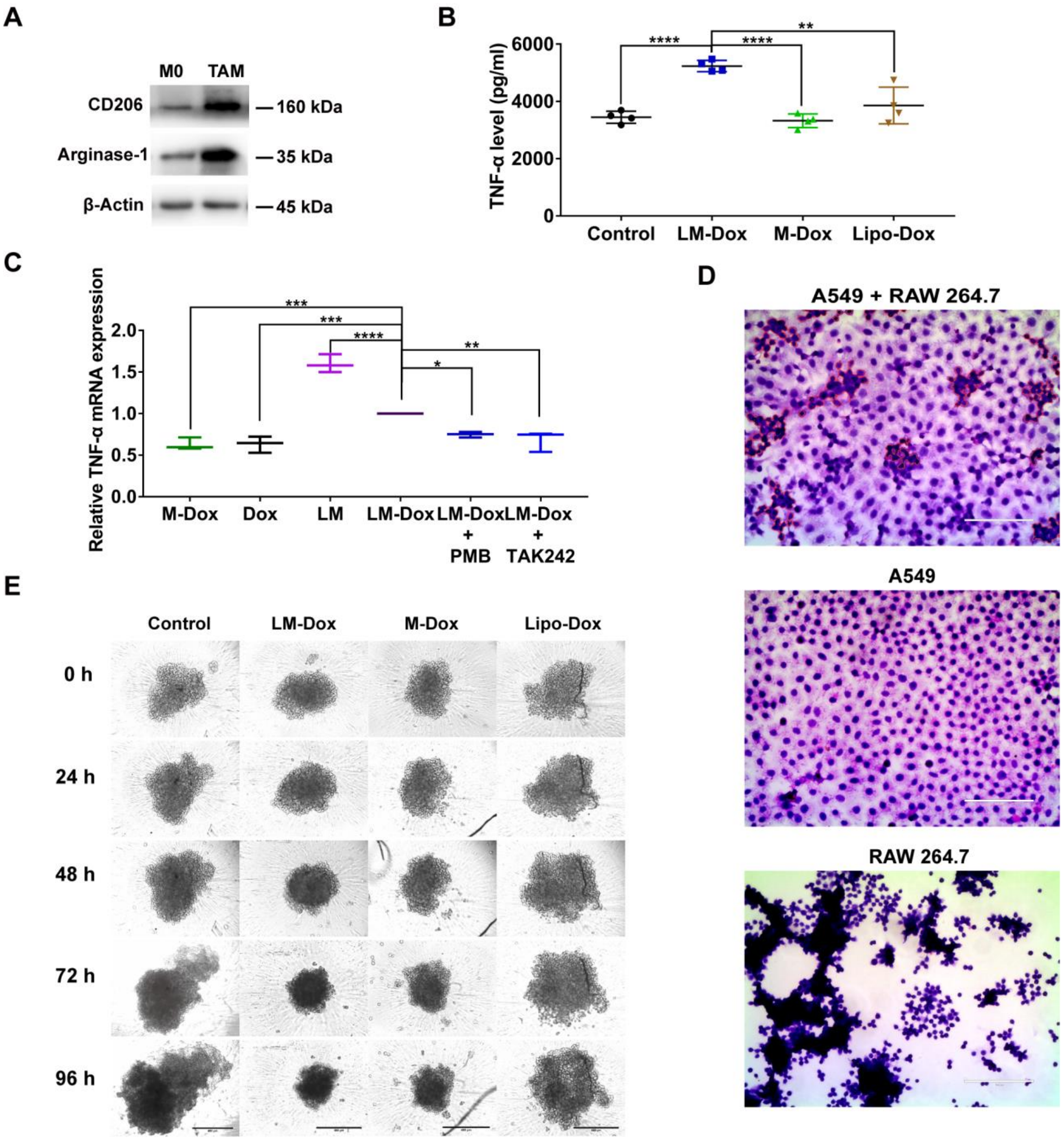

Figure S6. (A) Western blot images in RAW264.7 stimulated with A549 tumor cell conditioned medium for 2 days. Whole-cell extract was prepared and quantified. An equivalent amount of total protein was analyzed with antibodies against CD206, arginase-1 and β-Actin as the loading control. (B) ELISAs were performed to examine the production of TNF-α in the cell culture

supernatants. (C) The mRNA levels of TNF-α were analyzed by real-time qPCR. PMB was used to block the effect of LPS-induced activity. TAMs were pretreated with TAK242 before incubated (n = 3). (D) Representative images of A549 tumor cells and RAW264.7 macrophages stained with H&E. Cell boundaries of macrophages were marked with red lines. Scale bars, 100 μm. (E) The cytotoxic effects on the 3D tumor-immune spheroids. Microscopy images documenting the growth of representative spheroids after exposure to LM-Dox, M-Dox, Lipo-Dox from day 0 to day 4. Scale bars, 400 μm. The data are shown as mean ± s.d., * is $p < 0.05$, ** is $p < 0.01$, *** is $p < 0.001$, **** is $p < 0.0001$ by one-way ANOVA test.

The dual culture experiments were performed to evaluate whether LPS was temporarily attached to the cell membrane (receptors) of macrophages. Transwell was utilized a physical barrier (0.4 μm) to separate the FITC-labeled LPS anchored LM-Dox (upper chambers) and unmodified macrophages (lower chambers) while allowing to share the diffusible materials including free LPS (Figure S6A-B). An equal amount of free FITC-labeled LPS and macrophages without LPS modification were used as controls. After 24h of dual culture, free LPS in the upper chambers crossed the transwell membrane freely and was bound to the macrophages resulting in a significant increase in fluorescence intensity of the macrophages in the lower chamber. LM-Dox with FITC-labeled LPS attached to the cell membrane in the upper chamber did not enhance the fluorescence intensity of the macrophages in the lower chamber compared with that in the control groups suggesting that LPS anchored on the cell surface of LM-Dox is bound in a relatively stable manner.

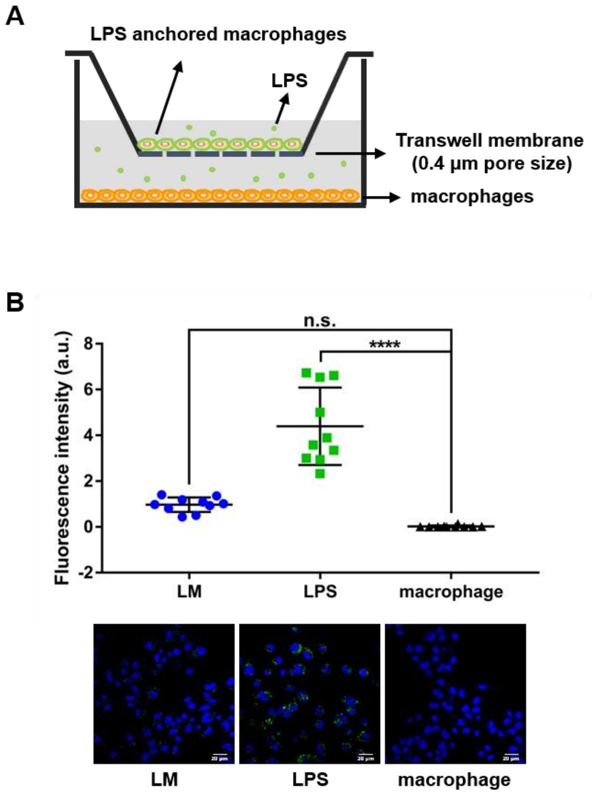

Figure S7. (A) A schematic depicting the cell dual-culture. FITC-LM (FITC-labeled LPS), FITC-labeled LPS or macrophages were cultured in the upper chamber of transwells, and macrophages were plated in the bottom chamber. (B) LPS-FITC on macrophages in bottom chamber was imaged by CLSM at 24 h. The nuclei were stained with DAPI. LPS-FITC produced a green fluorescence. The merged images were the overlay of three individual images. Scale bar, 20 μm. The data are shown as mean ± s.d., n = 10 per group, **** is $p < 0.0001$ by one-way ANOVA test.
